# Synthetic rescue in *Saccharomyces cerevisiae*: Concepts, large-scale genetic mapping, and functional implications

**DOI:** 10.71150/jm.2512017

**Published:** 2026-03-12

**Authors:** Ji Eun Choi, Woo-Hyun Chung

**Affiliations:** College of Pharmacy, Duksung Women’s University, Seoul 01369, Republic of Korea

**Keywords:** synthetic rescue, genetic interaction networks, *Saccharomyces cerevisiae*, systems biology, computational prediction, network robustness

## Abstract

Synthetic rescue (SR) describes a genetic interaction in which the deleterious effect of a primary mutation is compensated by a second mutation, restoring cellular function or viability. In *Saccharomyces cerevisiae*, SR complements synthetic lethality (SL) by revealing compensatory mechanisms that maintain essential biological processes. Classical studies established SR as a fundamental principle of genetic robustness in yeast. Subsequent development of high-throughput genetic tools, including Synthetic Genetic Array (SGA), Epistatic Miniarray Profile (E-MAP), and CRISPR interference (CRISPRi), has enabled systematic identification of SR interactions across pathways of genome maintenance, proteostasis, and metabolism. Integration of these experimental datasets with computational and network-based analyses has transformed SR research from descriptive genetics into a predictive framework. Databases such as BioGRID, TheCellMap, and Mslar further support SR inference and link yeast genetic networks to human disease models. Understanding SR has important translational implications. The same compensatory logic that restores viability in yeast can explain therapeutic resistance in cancer cells. Together, these insights reveal SR as a powerful concept connecting microbial genetics with systems medicine, emphasizing that robustness and resilience are dynamic properties of living systems.

## Introduction

Cellular processes are governed not by individual genes acting in isolation, but through extensive genetic interactions that form interconnected networks. Genes involved in DNA replication, repair, metabolism, transcription, and signaling pathways coordinate to maintain cellular homeostasis. The functional output of a cell is therefore an emergent property of these networks, rather than the sum of individual gene functions (Boone et al., 2007).

Perturbation of one gene can propagate through its network, producing context-dependent outcomes. In some cases, the loss of one pathway is buffered by compensatory mechanisms in parallel pathways, preventing severe phenotypes and ensuring robustness of the system (Hartman et al., 2001). For example, DNA damage repair relies on multiple partially redundant mechanisms, such that defects in one repair factor may be tolerated if alternative pathways remain functional (Boiteux and Jinks-Robertson, 2013; Prakash et al., 1993; Symington and Gautier, 2011). Conversely, when two genes that act in parallel or overlapping processes are simultaneously perturbed, the combined defect can uncover hidden vulnerabilities and lead to profound cellular dysfunction (Costanzo et al., 2010). This interdependence is not limited to DNA repair; it is a general property of biological systems, spanning processes such as metabolic flux, protein folding, and signal transduction (Giaever et al., 2002).

Such phenomena demonstrate that the functional state of a gene cannot be fully understood outside of its network context. Interactions among genes shape phenotypic variability, modulate disease susceptibility, and define potential targets for therapeutic intervention (Boone et al., 2007; Hartman et al., 2001). The study of genetic interactions therefore provides not only a map of cellular organization but also a framework for predicting how complex traits and pathological states emerge from molecular networks (Costanzo et al., 2010).

The concept of synthetic lethality (SL) was first described in classical genetics as a genetic interaction in which two individually viable mutations, when combined, lead to cell death (Dobzhansky, 1946). In *Saccharomyces cerevisiae*, systematic genetic interaction mapping enabled the identification of thousands of SL and synthetic sick (SS) pairs, laying the foundation for functional genomics. The SGA technology enabled high-throughput genetic interaction mapping in yeast, revealing extensive interaction patterns that clustered within biological pathways (Tong et al., 2004). This effort was expanded in a landmark study that screened over 5.4 million double mutants among 1,712 genes, constructing a global genetic interaction map of the yeast cell. These studies established that negative genetic interactions, including both SL and SS, are strongly enriched within functional modules, highlighting the modular and redundant architecture of cellular networks (Costanzo et al., 2010).

The biomedical impact of SL is exemplified by its translation to precision oncology. Tumors deficient in *BRCA1* or *BRCA2*, which compromise homologous recombination (HR) repair, are exquisitely sensitive to poly(ADP-ribose) polymerase (PARP) inhibitors such as olaparib (Bryant et al., 2005; Czyż et al., 2016; Farmer et al., 2005). This discovery led to FDA-approved targeted therapies that selectively kill *BRCA*-mutant cancer cells while sparing normal tissues. Beyond PARP, checkpoint kinase inhibition has emerged as another SL strategy. CHK1 inhibition synergizes with defects in the ATR–p53 axis, leading to selective sensitivity of p53-deficient cells to DNA-damaging agents (Nghiem et al., 2001). Similarly, inhibition of WEE1 kinase by MK-1775 (adavosertib) sensitizes p53-deficient tumors to chemotherapy, exemplifying how kinase inhibitors can exploit genetic vulnerabilities (Hirai et al., 2009). These examples demonstrate how genetic interaction principles uncovered in *S. cerevisiae* have provided a conceptual and methodological foundation for understanding disease networks and developing targeted therapies in humans (Boone et al., 2007; Costanzo et al., 2016). Complementarily, the synthetic genetic principles established in yeast continue to serve as a translational model for identifying therapeutic vulnerabilities and drug–gene interactions in human cancer systems (Choi and Chung, 2019).

**Fig. 1.**
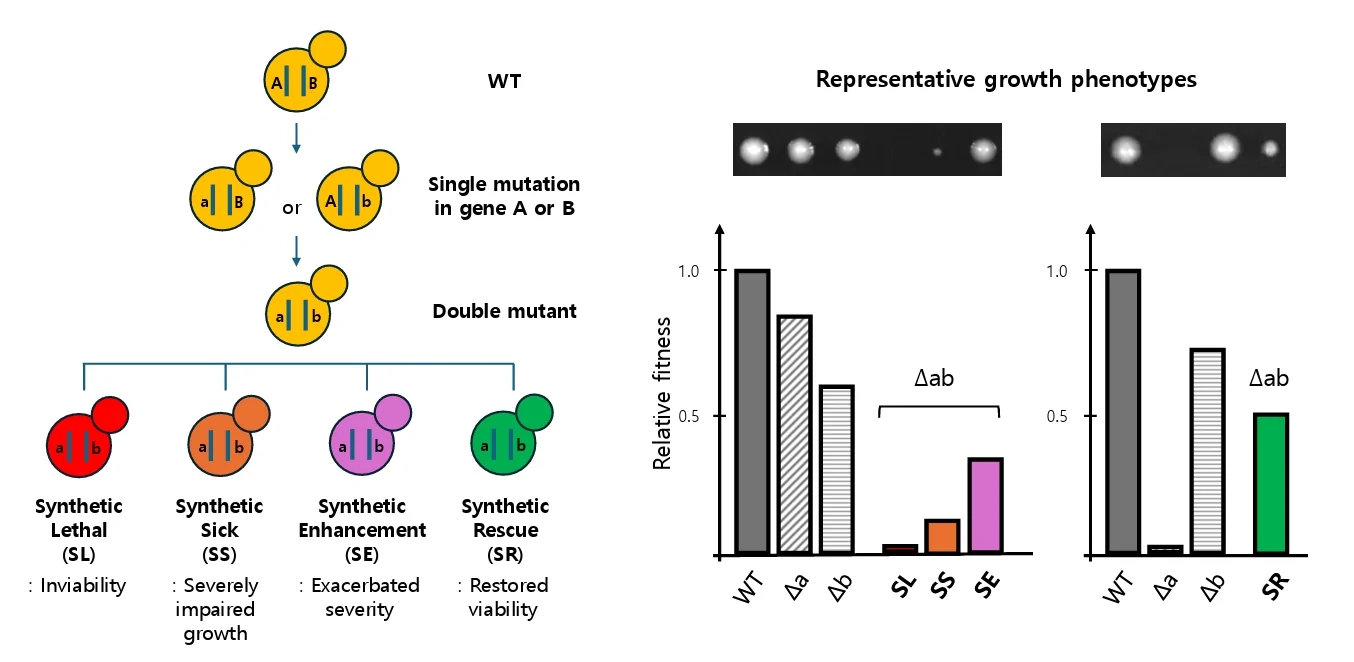
Schematic overview of genetic interaction outcomes in *S. cerevisiae*. Starting from a wild-type genotype (A|B), single mutations in either gene (a|B or A|b) reduce fitness or growth. When both mutations are combined (a|b), the resulting double mutant exhibits one of four outcomes: synthetic lethality (SL, red; inviability), synthetic sickness (SS, orange; severely impaired growth), synthetic enhancement (SE, purple; exacerbated severity), or synthetic rescue (SR, green; restored viability). These interactions are visualized via representative growth phenotypes (top images) and quantified as relative fitness (bottom bar graphs). This conceptual diagram illustrates how the combination of mutations can lead to either network collapse or compensatory recovery, revealing the dual nature of fragility and robustness in genetic systems. The relative fitness scale represents the normalized growth phenotype (typically colony size or exponential growth rate) relative to the wild type (set to 1.0). A fitness value of 0 indicates lethality, while 1 indicates wild-type growth.

Beyond lethality, other classes of genetic interactions broaden our understanding of network robustness. SS captures cases where combined mutations impair but do not abolish fitness, providing nuanced insights into buffering capacity (Costanzo et al., 2010). Synthetic enhancement (SE) describes situations in which a mutation in one gene exacerbates the severe phenotype of a mutation in another gene, revealing functional redundancies and parallel pathways (Boone et al., 2007; Guarente, 1993). Together, SL, SS, and SE illustrate the spectrum of negative genetic interactions that map the fragility of cellular systems. In contrast, compensatory interactions that alleviate deleterious phenotypes synthetic rescue (SR) highlight the opposite property: the resilience and adaptability of biological networks. These four interaction types are schematically illustrated in Fig. 1, highlighting the continuum from network fragility to compensatory resilience.

## Defining Synthetic Rescue Conceptual Framework

SR describes a genetic interaction in which the deleterious effect of a primary mutation is mitigated by a second mutation, thereby restoring viability or cellular function. Unlike SL, which exposes vulnerabilities by combining mutations that are individually tolerated but lethal together, SR uncovers compensatory mechanisms that emphasize the resilience of biological networks (Boone et al., 2007; Klein, 2000) (Fig. 1). However, SR must be distinguished from general buffering; while buffering reflects a system’s intrinsic capacity to passively absorb perturbations through pre-existing redundancy, SR represents a distinct compensatory mode in which a secondary mutation actively alleviates the deleterious effects of a primary defect. Furthermore, the definition of SR is not limited to the complete restoration of wild-type fitness; it encompasses specific mechanistic recovery, as opposed to non-specific growth variation, since these partial rescue events often provide critical insights into metabolic rewiring and the emergence of drug resistance.

SR interactions can be broadly categorized based on their genetic and mechanistic features. Intragenic SR occurs when two mutations within the same gene or closely linked locus counteract one another. A well-documented case is the β-tubulin mutant *tub2-406*, in which several second-site suppressor mutations were mapped directly to the *TUB2* locus, thereby restoring microtubule function (Pasqualone and Huffaker, 1994). In contrast, intergenic SR arises when mutations in distinct genes compensate for each other. The same study identified STU1, encoding a spindle-associated protein, as an extragenic suppressor of *tub2* mutants, illustrating cross-gene compensation within the microtubule assembly pathway (Pasqualone and Huffaker, 1994). Another intergenic example comes from the HR network, where the synthetic lethality of *srs2* *rad54* double mutants is suppressed by mutations in *RAD51*, *RAD52*, *RAD55*, or *RAD57* (Klein, 2000).

A second classification distinguishes between complete and partial rescue. Complete rescue refers to cases in which the secondary mutation restores growth or viability close to wild-type (WT) levels. The severe growth defects of *top3* mutants are almost fully alleviated by deletion of *SGS1*, highlighting robust intrapathway compensation (Wagner et al., 2006; Weinstein and Rothstein, 2008). By contrast, partial rescue describes improvements that do not reach WT performance but still mitigate deleterious phenotypes. For example, metabolic rewiring in *zwf1* *lsc2* double mutants enhances growth relative to *zwf1* single mutants, but restoration remains incomplete, underscoring the biological significance of even partial rescue (Partow et al., 2017).

Finally, SR can be classified as direct or indirect rescue. Direct rescue occurs when the secondary mutation acts within the same molecular process, such as loss of *SRS2* suppressing the UV sensitivity of *rad6* mutants by channeling DNA lesions into the *RAD52* recombination pathway (Schiestl et al., 1990). Indirect rescue, by contrast, is mediated through network-level adaptations, such as the metabolic bypass that enables *zwf1* *lsc2* cells to reconfigure redox homeostasis via alternative routes (Partow et al., 2017). These distinctions emphasize the mechanistic diversity of SR and its utility as a framework for dissecting genetic robustness.

## Experimental Dissection of Synthetic Rescue Interactions in Yeast

SR interactions were initially discovered through classical genetic approaches in *S. cerevisiae*. In early studies, targeted gene deletions were utilized to identify secondary mutations that could alleviate the deleterious phenotypes caused by primary loss-of-function mutations. These pioneering efforts established the conceptual foundation for SR by demonstrating that essential cellular processes could be restored through compensatory genetic changes.

One of the earliest documented intra-pathway cases involved the DNA topological processing pathway. Mutation of *SGS1*, encoding a RecQ-family helicase, was shown to alleviate the slow-growth and genome-instability phenotypes of *top3* mutants, highlighting a compensatory balance within the Sgs1-Top3 complex (Gangloff et al., 1994). This interaction underscores the importance of protein complex stoichiometry; the rescue occurs not by restoring Top3 activity, but by preventing the Sgs1-dependent accumulation of toxic recombination intermediates (e.g., Holliday junctions) that remain unresolved in the absence of Top3 (Fabre et al., 2002; Gangloff et al., 1994). Interestingly, Sgs1 also engages in SE interactions: combined defects of *sgs1* *dna2-1* produce severe genome instability, exemplifying how the same gene can participate in both rescue and aggravating genetic outcomes depending on its partner (Zhu et al., 2008). This contrast underscores the mechanistic diversity of genetic interactions, where SR and SE represent two sides of pathway interdependence.

Similarly, within the DNA damage–tolerance network, loss of *SRS2* function was found to suppress the pronounced UV sensitivity of *rad6* and *rad18* cells. This exemplifies a mechanism based on DNA repair pathway choice: Srs2 normally inhibits HR to prevent inappropriate recombination events. Its absence lifts this inhibition, allowing the HR machinery to salvage replication gaps left unrepaired by the defective Rad6/Rad18 post-replication repair pathway (Krejci et al., 2003). Consistent with this mechanism, this rescue required intact components of the *RAD52* recombination pathway, underscoring that SR often operates by channeling lesions into alternative repair mechanisms (Palladino and Klein, 1992; Schiestl et al., 1990). Building on these findings, subsequent work revealed SR events within the HR module. The SL of *srs2* *rad54* double mutants was suppressed by mutations in *RAD51*, *RAD52*, *RAD55*, or *RAD57*, further illustrating that SR interactions frequently occur among functionally interconnected genes within the same pathway (Klein, 2000; Palladino and Klein, 1992). Collectively, these observations revealed that SR events frequently occur within the same biological pathway or compensatory module (Boone et al., 2007).

**Fig. 2.**
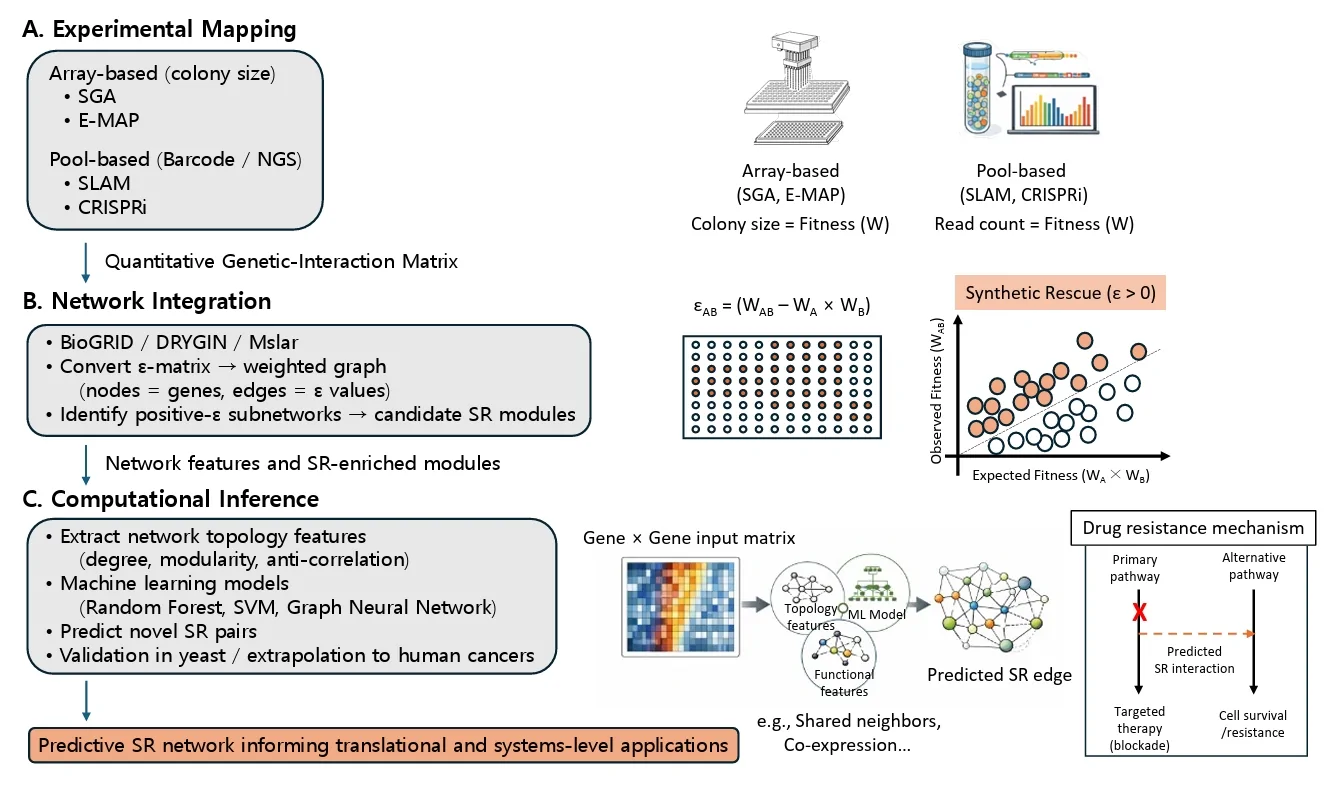
Integrated experimental, network, and computational pipeline for SR discovery. (A) Experimental Mapping: High-throughput phenotyping is categorized into array-based methods (SGA, E-MAP) which measure colony size as a proxy for fitness, and pool-based methods (SLAM, CRISPRi) which quantify strain abundance via NGS barcodes. (B) Network Integration: Fitness data is transformed into genetic interaction scores (ε) based on the multiplicative model (ε_AB_ = W_AB_ - W_A_ × W_B_). The scatter plot illustrates SR interactions (orange points) where the observed double-mutant fitness (W_AB_) significantly exceeds the expected fitness (W_A_ × W_B_). These scores are integrated with curated databases (e.g., BioGRID, Mslar) to identify SR-enriched subnetworks. (C) Computational Inference: Machine-learning models, trained on network topology (e.g., shared neighbors) and functional features (e.g., co-expression), predict novel SR edges. This results in a predictive framework for translational models, illustrating how SR interactions act as alternative pathways to bypass targeted therapy blockades and confer drug resistance.

Subsequent advances in functional genomics enabled more systematic discovery of SR interactions. The construction of genome-wide deletion libraries, such as the yeast knockout (YKO) collection, allowed for high-throughput interrogation of genetic interactions using Synthetic Genetic Array (SGA) technology (Tong et al., 2004). SGA automates the mating, sporulation, and haploid selection steps, producing large collections of double mutants that can be quantitatively assayed for fitness. While originally designed to map SL interactions, its quantitative extensions such as Epistatic Miniarray Profiles (E-MAP) provided continuous interaction scores rather than binary lethal/viable calls, thereby uncovering intermediate phenotypes such as partial rescue or buffering effects (Schuldiner et al., 2005) (Fig. 2).

Building on this, Synthetic Lethality Analysis by Microarray (SLAM) exploited random spore generation combined with microarray-based barcode hybridization, enabling researchers to track the relative abundance of thousands of double mutants simultaneously in a pooled format. This increased throughput allowed detection of both negative (synthetic sick/lethal) and positive (rescue) interactions across the genome (Ooi et al., 2006).

In parallel, random spore analysis itself served as a foundational strategy for double mutant construction. By sporulating heterozygous diploids and recovering haploid progeny carrying both mutations, investigators could rapidly generate and score large numbers of genetic combinations. When coupled with barcode-based readouts, random spore methods became especially powerful for capturing context-dependent SR phenotypes in parallel (Pan et al., 2006).

Through SGA and its derivatives, large-scale double mutant collections could be quantitatively evaluated for phenotypic restoration. One illustrative example involves *zwf1* mutants, which exhibit impaired NADPH biosynthesis due to disruption of the oxidative pentose phosphate pathway. When combined with *lsc2*, which alters TCA-cycle succinyl-CoA flux and rebalances cellular redox metabolism, the double mutant displays significant growth recovery, representing a form of SR mediated by metabolic rewiring (Partow et al., 2017). Additional SGA-based studies have reported similar metabolic and stress-related rescues, reinforcing the idea that SR is not an isolated phenomenon but a pervasive feature of cellular networks.

In addition to full gene deletions, the decreased abundance by mRNA perturbation (DAmP) strategy has been developed to enable partial knockdown of essential genes. In this approach, destabilizing sequences are inserted into 3' untranslated regions (3' UTR) to reduce transcript stability and protein expression without complete gene loss. DAmP strains are particularly useful for identifying SR interactions involving essential genes that cannot be deleted (Breslow et al., 2008). More recently, CRISPR-based platforms including CRISPR interference (CRISPRi) have facilitated tunable modulation of gene expression in yeast (McGlincy et al., 2021). These approaches are particularly valuable when gene deletion leads to lethality or pleiotropic effects. In parallel, pooled mutant libraries tagged with DNA barcodes have been applied in growth competition assays under diverse stress conditions such as oxidative stress or nutrient limitation, revealing condition-specific SR interactions at genome-wide scale (Jaffe et al., 2017).

Taken together, experimental efforts in yeast have shown that SR interactions are not limited to static genetic redundancies, but instead reflect dynamic and context-dependent reorganization of cellular networks. These findings highlight the utility of *S. cerevisiae* as a model system for dissecting the molecular architecture of SR and exploring its conservation across eukaryotes.

## Computational Inference and Prediction of Synthetic Rescue Interactions

With the increasing complexity of genetic interaction networks, computational approaches have become indispensable for the systematic discovery of SR interactions. In *S. cerevisiae*, the accumulation of genome-scale knockout, perturbation, and quantitative interaction datasets—such as the YKO collection, SGA maps, and E-MAPs has provided a foundation for computational inference of SR. These resources have enabled statistical, network-based, and machine learning frameworks to predict rescue relationships from high-dimensional data.

Quantitative assessment of genetic interactions began with the E-MAP approach, which measured double-mutant growth phenotypes relative to corresponding single mutants (Schuldiner et al., 2005). This approach introduced a quantitative definition of the epistasis score (ε), based on the multiplicative model which posits that the expected fitness of a double mutant is the product of single mutant fitness values (W_A_ × W_B_). The score represents the deviation between observed double-mutant fitness (W_AB_) and this expectation (ε_AB_ = W_AB_ - W_A_ × W_B_) (Collins et al., 2006). While this framework provides a robust metric for global mapping, it assumes functional independence between non-interacting genes and can be susceptible to experimental noise or floor effects, particularly when assessing strains with severe growth defects. A negative ε value indicates synthetic sickness or lethality, whereas a positive ε signifies buffering or rescue. However, simple ε scoring fails to capture the directionality and context dependence of SR, prompting the development of higher-order computational models integrating network topology and conditional information (Fig. 2).

Network-based approaches prioritize candidate SR interactions using topological signals such as modular buffering and pathway redundancy, and integrate curated resources including BioGRID, TheCellMap, Mslar, STRING, and GeneMANIA to support inference from high-dimensional genetic interaction data (Costanzo et al., 2016; Oughtred et al., 2021; Szklarczyk et al., 2023; Warde-Farley et al., 2010; Zhu et al., 2023).

A growing number of databases have facilitated network-based prediction by providing curated and quantitative genetic interaction information. BioGRID integrates physical and genetic interactions, including SR annotations from yeast high-throughput screens (Oughtred et al., 2021). DRYGIN and TheCellMap, derived from SGA datasets, offer probabilistic interaction scores and visualization tools that help identify positive genetic interactions (Costanzo et al., 2010, 2016). In practice, DRYGIN-derived epistasis matrices have been mined to identify subnetworks enriched for positive ε values, revealing compensatory modules in DNA replication and mitochondrial maintenance. Integrating BioGRID and Mslar datasets further enabled the construction of supervised classifiers that successfully recovered known SR pairs and predicted novel candidates across yeast metabolism and DNA repair pathways (Oughtred et al., 2021; Zhu et al., 2023). Mslar, in particular, serves as a primary microbial benchmark set for evaluating algorithmic precision in yeast. Alongside these interaction datasets, STRING and GeneMANIA enhance SR inference through functional-association evidence such as co-expression or shared pathways (Szklarczyk et al., 2023; Warde-Farley et al., 2010). Expanding the scope to human biology, SynLethDB distinguishes itself by aggregating experimentally supported and literature-curated synthetic-lethal interactions specifically in human cells with its 2.0 knowledge-graph update enabling cross-species reasoning that informs SR modeling in cancer contexts (Guo et al., 2016; Wang et al., 2022) (Fig. 2).

Building on these resources, machine-learning (ML) approaches have been applied to systematically predict SR pairs using features such as gene essentiality, co-expression, co-functionality, and network proximity. Graph-based models trained on TheCellMap interaction graphs have predicted unobserved SR edges, many of which were subsequently validated in pooled CRISPRi screens (McGlincy et al., 2021). Supervised classifiers, including random forests and support-vector machines, have achieved high recall for known SR pairs and expanded the candidate space to previously uncharacterized modules. More recently, graph-embedding and deep-learning frameworks have leveraged topological information to improve cross-species prediction accuracy. Transfer-learning approaches have further enabled yeast-trained models to forecast SR relationships in human cancer cell lines, where secondary mutations often restore viability after therapeutic inhibition (Fig. 2).

Computational frameworks have also been applied to translational medicine, revealing clinically relevant synthetic interactions. For instance, SL prediction models have identified anticancer targets such as *BRCA*–PARP and *ARID1A–ATR*, which led to selective therapies exploiting DNA repair defects (Bryant et al., 2005; Farmer et al., 2005; Williamson et al., 2016). Similarly, SR-based modeling has illuminated mechanisms of drug resistance, including HUWE1-mediated restoration of *BRCA1*–*RAD51* repair conferring PARP-inhibitor resistance, and compensatory PI3K/AKT reactivation that offsets MEK inhibition in *KRAS*-driven cancers (Pettitt et al., 2023; Roper et al., 2014). These examples highlight how SR and SL predictions bridge yeast genetic principles with precision oncology.

By integrating experimental and computational perspectives, these approaches have transformed SR discovery from a serendipitous observation into a predictive, network-informed discipline. They not only accelerate hypothesis generation but also bridge fundamental yeast genetics with translational applications, such as forecasting drug-resistance mechanisms, identifying compensatory modules in cancer cells, and designing synthetic pathways for metabolic engineering. An integrated overview of these experimental and computational approaches is summarized in Fig. 2, which outlines how genetic perturbation data, network integration, and predictive modeling converge to uncover SR interactions in yeast and beyond.

## Functional and Translational Implications

SR interactions reveal how cellular networks adaptively reorganize to maintain viability under genetic or environmental perturbations. In *S. cerevisiae*, SR mapping exposes the modular logic of genome stability, proteostasis, and metabolic balance, demonstrating that rescue is not a random occurrence but a structured response of the network. Rather than relying on singular suppressor mutations, yeast studies indicate that SR operates through distinct topological principles, such as the activation of parallel pathways to bypass metabolic blockades and the functional substitution of DNA repair modules. Furthermore, at the protein homeostasis level, *HSP104* overexpression rescues folding defects in *sba1* and *hsp90* mutants, underscoring the buffering capacity of proteostasis networks (Nathan et al., 1997). Collectively, these findings highlight that SR reflects an inherent self-stabilizing property of biological systems.

The translational significance of SR extends to human disease, particularly in elucidating the mechanisms of adaptive therapeutic resistance. Analogous to genetic buffering in yeast, tumor cells frequently exploit rescue pathways to evade the lethal effects of targeted inhibitors. For instance, *BRCA1*-deficient cancers acquire PARP-inhibitor resistance via HUWE1-mediated stabilization of *BRCA1*-Δ11q or *RAD51* upregulation (Pettitt et al., 2023), while *KRAS*-mutant colorectal cancers reactivate PI3K/AKT signaling after MEK inhibition, forming a rescue circuit that can be disrupted by combined pathway blockade (Roper et al., 2014). These examples illustrate that SR not only reveals network robustness but also explains adaptive failure modes in therapy.

Thus, insights from yeast SR networks translate into predictive frameworks for human biology, mapping how gene and drug interactions create either vulnerability or resilience. Integrating high-throughput genetics with computational prediction will be key to identifying network states that toggle between synthetic lethality and rescue, offering a foundation for both precision oncology and rational design in synthetic biology. The principal experimental and computational methodologies that have enabled SR discovery are summarized in Table 1, providing a comparative overview of techniques, key outputs, and representative references.

## From Yeast Networks to Predictive Medicine: Future Directions in Synthetic Rescue Research

The field of SR stands at a critical juncture. After decades of genetic dissection in *S. cerevisiae*, the phenomenon once viewed as an incidental suppressor effect now represents a unifying principle of biological resilience (Boone et al., 2007). Yet the scope of SR remains unevenly charted compared with SL, largely because its defining feature, the restoration of fitness, is inherently conditional and context-dependent (Lehner, 2011). Future research must therefore move beyond cataloging suppressor pairs to establishing predictive frameworks that can explain when, how, and why rescue emerges.

Technological advances are beginning to bridge this gap. Next-generation phenomics, enabled by CRISPR-based perturbation and automated growth profiling, allows systematic quantification of partial and dynamic rescue events (McGlincy et al., 2021). Time-resolved fitness assays combined with transcriptomic and proteomic readouts could uncover the temporal choreography of compensation, revealing how gene networks reconfigure in real time following perturbation (Schuldiner et al., 2006). Progress in single-cell sequencing and lineage tracing now makes it possible to observe SR at the level of individual cells, distinguishing transient buffering from stable rewiring. The integration of these approaches promises to reveal not only which mutations rescue but also how they act within the architecture of cellular regulation (Costanzo et al., 2016).

At the computational level, SR prediction is entering an era of data-driven synthesis. Traditional epistasis scoring, while foundational, provides only a static snapshot of interaction strength. The next step is to embed SR into dynamic network models where interaction strength evolves with context (Barabási and Oltvai, 2004). Graph-learning algorithms and probabilistic network inference can combine genetic, expression, and metabolomic data to identify compensatory motifs that anticipate rescue behavior (Zhu et al., 2023). Training these models across species, using yeast as the baseline and mammalian systems as validation, will reveal conserved patterns of adaptability. Such cross-domain learning can ultimately convert SR mapping from descriptive genetics into predictive network medicine, capable of forecasting how a cell or tumor will reorganize in response to stress or therapy (Rancati et al., 2018).

Finally, SR research invites a conceptual reorientation of systems biology itself. Rather than viewing robustness as a passive buffer against failure, SR reframes it as an active process of reorganization (Boone et al., 2007; Lehner, 2011). Understanding rescue means understanding how networks adapt, not only in response to genetic mutation but also across environmental, developmental, and therapeutic contexts. In this view, SR becomes a model for resilience at all scales, from molecular circuits and metabolic pathways to evolutionary and ecological systems. The ultimate goal is not merely to catalogue instances of compensation but to articulate the general principles by which complex systems sustain function under constraint (Barabási and Oltvai, 2004; Rancati et al., 2018).

By uniting experimental genomics, computational modeling, evolutionary analysis, and translational design, the study of SR in *S. cerevisiae* will continue to illuminate the universal logic of adaptation. From yeast colonies evolving under selective stress to human cells evading pharmacologic blockade, the same rules of network recovery apply. Charting these rules will define the next decade of SR research and may establish a new synthesis between genetics and systems medicine, where robustness is not the opposite of fragility but its necessary complement.

## Conclusion

Across decades of yeast genetics, SR has revealed that cellular networks are not static architectures but self-correcting systems capable of restoring equilibrium after disruption. The same principles that enable *S. cerevisiae* to regain growth after loss of function in essential genes also illuminate how human cells survive under therapeutic stress. In this sense, SR bridges two domains that have long been studied separately: the molecular logic of robustness and the evolutionary logic of survival.

The core lesson from yeast is that compensation is not accidental. It arises from the redundancy, modularity, and dynamic connectivity of genetic networks. When one route fails, another can rewire to restore balance. Such flexibility is what allows cells to maintain viability despite thousands of possible perturbations. Early studies of intra-pathway rescue, such as suppression of *top3* mutants by loss of *SGS1* or rescue of *rad18* by *srs2* null mutation, demonstrated that suppression follows recognizable biochemical logic rather than random chance. The accumulation of high-throughput data, from SGA to CRISPR interference libraries, has transformed this logic into quantifiable network principles. Through systematic mapping, yeast has shown that rescue is distributed non-randomly across biological modules, revealing a hidden structure of compensatory potential that parallels how diseases adapt and resist therapy in higher organisms.

The translational significance of SR lies in its emerging predictive potential. In oncology, SR logic offers a promising framework for two complementary strategies. First, anticipating resistance: if loss of one gene induces dependence on another, then identifying those compensatory dependencies could reveal where resistance will emerge. Second, inducing protection: in degenerative or metabolic diseases, artificially promoting SR-like states might restore function by stabilizing fragile pathways. Both approaches require accurate prediction of network-level responses, a goal that is becoming increasingly feasible through integration of yeast-derived genetic interaction data, human CRISPR screens, and machine-learning inference (McGlincy et al., 2021; Zhu et al., 2023). However, translating these predictions into clinics requires rigorous validation, as off-target effects and the tissue-specific wiring of human networks pose significant hurdles to identifying safe therapeutic windows.

Looking forward, three priorities define the next phase of SR research. The first is mechanistic depth: identifying molecular intermediates and regulatory nodes that mediate rescue, moving beyond correlative network links to causal explanations. The second is computational generalization: developing machine-learning models that are transferable across organisms and conditions, capturing the shared grammar of compensation from yeast to human. The third is translational realization: designing interventions that can intentionally harness or inhibit SR pathways for therapeutic benefit. Achieving these goals will require interdisciplinary collaboration among geneticists, computational biologists, and clinicians, unified by the recognition that robustness and fragility are two sides of the same biological coin.

Ultimately, the legacy of yeast genetics lies not merely in mapping interactions but in revealing the logic by which life adapts. SR gives a summary of this logic: disruption followed by reorganization, loss followed by recovery, fragility balanced by resilience. As the boundaries between experimental genetics and predictive medicine become less clear, SR stands as a conceptual and practical bridge between them. By continuing to explore how cells restore what has been damaged, we may learn not only how to treat disease but also how to understand life itself, as a system that is always moving between collapse and renewal.

## Figures and Tables

**Table 1.** Summary of experimental and computational approaches for discovering SR interactions in *S. cerevisiae*

Category	Method / Resource	Core concept	Representative output	References
Experimental	SGA	High-throughput construction of double mutants and quantitative fitness scoring	Genome-wide ε-matrix defining SL, SS, and SR interactions	Costanzo et al. (2010); Tong et al. (2004)
	E-MAP	Quantitative measurement of genetic epistasis (ε)	Continuous distribution of interaction strengths	Schuldiner et al. (2005)
	SLAM	Barcode-based pooled analysis of double mutants	Parallel detection of SL and SR phenotypes	Ooi et al. (2006)
	Random-spore analysis	Haploid segregation of heterozygous diploids to generate double mutants	Context-specific rescue phenotypes	Pan et al. (2006)
	CRISPRi / DAmP perturbation	Tunable partial knockdown of essential genes	Identification of SR interactions involving essential loci	Breslow et al. (2008); McGlincy et al. (2021)
Computational	DRYGIN / TheCellMap	Probabilistic genetic-interaction matrices enabling network analysis	Visualization and clustering of positive ε modules	Costanzo et al. (2016)
	BioGRID / Mslar / STRING	Curated databases integrating physical and genetic associations	Functional-association networks supporting SR inference	Oughtred et al. (2021); Szklarczyk et al. (2023)
	Machine-learning models	Prediction based on essentiality, co-expression, and network topology	Computationally predicted SR pairs validated experimentally	Zhu et al. (2023)
	Translational modeling	Cross-species extension of SR/SL logic to disease and therapy	Prediction of drug resistance mechanisms: Reversion mutations in BRCA-deficient cancers, Adaptive bypass in *KRAS*-driven tumors	Roper et al. (2014); Williamson et al. (2016)
